# Potential side effect of propofol and sevoflurane for anesthesia of anti-NMDA-R encephalitis

**DOI:** 10.1186/1471-2253-14-5

**Published:** 2014-01-16

**Authors:** François-Xavier Lapébie, Céline Kennel, Laurent Magy, Fabrice Projetti, Jérôme Honnorat, Nicolas Pichon, Philippe Vignon, Bruno François

**Affiliations:** 1 Intensive Care Unit, University Hospital Dupuytren, Limoges, France; 2 Department of Neurology, University Hospital Dupuytren, Limoges, France; 3 Pathology Laboratory, University Hospital Dupuytren, Limoges, France; 4 Centre for diagnosis and treatment of paraneoplastic neurological syndromes, Hospices Civils de Lyon, Centre de recherche en neurosciences de Lyon, Université Claude Bernard Lyon 1, Inserm 1028/CNRS 5292, Lyon, France; 5 Service de Réanimation Polyvalente, Centre hospitalier universitaire Dupuytren, 2, avenue Martin Luther King, 87042 Limoges, Cedex, France

**Keywords:** Encephalitis, General anesthesia, NMDA, Propofol, Sevoflurane

## Abstract

**Background:**

Many anesthetic drugs interact with the NMDA receptor and may therefore alter the clinical presentation of anti-NMDA-R encephalitis.

**Case presentation:**

A 24-year-old woman was admitted to hospital for decreased consciousness and hyperthermia. Cerebrospinal fluid analysis revealed lymphocytic pleocytosis, and elevated protein. Cultures were negative. Patient state worsened with agitation, facial dyskinesia, ocular deviation, and limb dystonia. Diagnosis of anti-NMDA-R encephalitis was evidenced by specific antibodies. High doses of methylprednisolone were administered. CT scan disclosed an ovarian teratoma and tumor resection was scheduled under anesthesia with propofol, sufentanil, atracurium and sevoflurane. Sedation after surgery was maintained with propofol. Rapidly after surgery, patient’s condition deteriorated with increase of dyskinesias, and two tonic-clonic generalized seizure events.

**Conclusion:**

In patients with anti-NMDA-R encephalitis, anesthesia using benzodiazepines, opiates and curares, which fail to interfere with the NMDA pathway, should be preferred.

## Background

Anti-N-methyl-D-aspartate receptor (anti-NMDA-R) encephalitis has become the second most frequent cause of immune-mediated encephalitis and probably remains underdiagnosed. Its pathogenesis is based on NMDA receptor blockade by patient’s auto-antibodies [1]. Many anesthetic drugs interact with the NMDA receptor and may therefore alter the clinical presentation of anti-NMDA-R encephalitis [2]. We herein describe a patient with documented anti-NMDA-R encephalitis whose symptoms dramatically worsened after a general anesthesia.

## Case presentation

A 24-year-old woman with no medical history was admitted to the hospital for decreased consciousness and hyperthermia. Two months earlier, her relatives noticed behavioral changes with marked anxiety and depressive mood. Cerebrospinal fluid (CSF) analysis revealed lymphocytic pleocytosis with 470 nucleated cells/mm^3^ (99% of lymphocytes) and elevated protein level (84 mg/dl). CSF cultures and PCR analysis for HSV and VZV were negative. Electroencephalogram (EEG) disclosed non-specific, slow activity in the fronto-temporal region. Brain magnetic resonance imaging (MRI) depicted a few FLAIR hyperintense signals in the white matter. Infectious encephalitis was first suspected and amoxicillin in conjunction with aciclovir were administered. Progressive neurologic deterioration led to transfer the patient to the intensive care unit (ICU). On admission, the patient was confused, agitated, with a Glasgow Coma Score of 12. She also exhibited facial dyskinesia, ocular deviation with ocular dipping, and limb dystonia (A video shows this more in details, see Additional file 1 which shows dyskinesias). The patient was ventilated. She received rocuronium bromide (4 mg) and midazolam (10 mg) for intubation and was maintained on a continuous propofol infusion (50 to 150 mg/h discontinuously) in order to reach a Richmond Agitation Sedation Scale between 0 and -3. Limbic encephalitis was highly suspected because of her young age, the lack of medical history, the clinical presentation and the absence of alternative etiology. Intravenous immunoglobulins (IVIg) were promptly initiated (0.4 g/kg per day for 5 days). The diagnosis of anti-NMDA-R encephalitis was confirmed by the presence of specific antibodies in the CSF, revealed by a semi-quantitative test cell based assay. Since the patient exhibited no clinical improvement, high doses of methylprednisolone were administered intravenously (1 g/day for 5 days). Body CT scan disclosed a right ovarian tumor (27 mm) consistent with a teratoma. Tumor resection was scheduled on Day 19. The patient was then sedated with propofol 50 mg/h. General anesthesia was induced with a combination of propofol (100 mg), sufentanil (20 μg) and atracurium (40 mg) and maintained with sevoflurane at 2.5% during 70 min. No complication occurred. The tumor was solid, including teeth and hair and the pathological diagnosis was mature teratoma without malignancy. Sedation after surgery was maintained: propofol was reintroduced 6 hours after the end of the procedure. Eight hours after surgery, the patient’s condition deteriorated with a marked increase in the frequency and amplitude of dyskinesias (a video shows this in more details; see Additional file 2 which shows worsened dyskinesias). Another 8 hours later a tonic-clonic generalized seizure event occurred. It resolved spontaneously within one minute. Propofol flow was 50 mg/h; infusion was stopped because of impaired consciousness. Levetiracetam was then started. Dyskinesias improved but propofol (50 mg/h) was reintroduced 14 hours later because of agitation. Two hours after propofol reintroduction, a second tonic-clonic generalized seizure event occurred which was treated with intravenous phenytoin. Propofol was maintained (80 mg/h), but dyskinesias increased. Eight hours after the second seizure propofol was finally stopped as a side effect was suspected. The neurologic status progressively improved but the patient could not talk. Accordingly, rituximab was administered as a second-line therapy (375 mg/m^2^ per week for 4 weeks). The patient was discharged from ICU on Day 36. At 3-month follow-up, the patient’s neurological status was slowly improving.

## Discussion and conclusions

Anti-NMDA-R encephalitis is identified among autoimmune limbic encephalitis since 2007 [1,3]. All autoimmune limbic encephalitis share the same clinical presentation with psychiatric symptoms (personality changes, irritability, depression and hallucinations), short-term memory loss, confusion, coma, seizures and autonomic instability [1,3]. CSF analysis frequently identifies a lymphocytic pleocytosis, and EEG typically depicts delta or theta activity, either diffuse or in fronto-temporal regions. Brain MRI abnormalities are less frequent but diffuse hyperintense signal of the white matter may be encountered, as in the present case. Anti-NMDA-R encephalitis typically occurs in young women (mean age, 23 years old). It is characterized by frequent movement disorders (dystonia and dyskinesia) and is commonly associated with ovary teratomas (54% of the cases) [3]. Immunotherapy of anti-NMDA-R encephalitis is initially based on steroids, and subsequently relies on intravenous immunoglobulin or plasma exchange. Cyclophosphamide and/or rituximab are second-line therapies. Early removal of an underlying tumor may be associated with a good prognosis: up to 80% of patients fully recover or have mild sequelae after tumor removal and first-line immunotherapy (corticosteroids and/or IVIg and/or plasma exchange) [1]. Nevertheless, the improvement is typically slow, over several months. The median time from symptom presentation to initial signs of improvement is 8 weeks (range 2–24 weeks) for patients with early tumor treatment [3]. Coma, autonomic instability and dyskinesias improve first, then social behavior and executive function [1]. We did not find any articles reporting a worsening of symptoms following resection. On the contrary a case of huge improvement within 48 hours after the operation is reported [4]. Rapid neurological response to tumor removal is however infrequent; it suggests that anesthetic drugs affect NMDA-R function [1].

NMDA-R, α-amino-5-methyl-3-hydroxy-4-isoxazole propionic acid receptor (AMPA-R) and kainate receptor are the 3 subtypes of ionotropic glutamate receptors. Anti-NMDA-R antibodies may be produced as a consequence of immunization against ectopic brain tissue found in teratoma [5] and induce glutamatergic transmission impairment. They bind to the NR1 subunit of the NMDA-R and disrupt surface cross-talk between NMDA and Ephrin-B2 receptors. This results in the displacement of surface GluN2A-NMDA receptors out of synapses, and leads to a selective and reversible decrease in NMDA-R surface density at the post-synaptic level of the glutamatergic synapse which correlates with antibody titers [6,7]. Inactivation of inhibitory GABAergic interneurons, which express high concentrations of NMDA-R, has also a key role in pathophysiology [1]. The result is a hyperglutamatergic state with an imbalance between NMDA and AMPA pathways [6].

Many anesthetic drugs inhibit NMDA-R and therefore can induce the same symptoms as those observed in anti-NMDA-R encephalitis [1]. NMDA-R are strongly inhibited by nitrous oxide, xenon and ketamine [8]. Although several authors emphasized the threat of potential worsening of symptoms after the use of these anesthetic drugs [2,9], no case has yet been reported to the best of our knowledge.

In our patient, the side effect of general anesthesia is presumably responsible for the clinical worsening which followed the teratoma removal. Propofol is a short-acting drug, administered intravenously, and this phenolic-derivative hypnotic agent produces general anesthesia by enhancing GABAergic transmission [9]. In vitro, propofol may also block NMDA-R at the concentrations routinely used to maintain a general anesthesia, by inhibiting the phosphorylation of NR1 subunit in neurons [10]. Another in vitro experiment showed that propofol inhibits NMDA-R through an allosteric modulation of channel gating but at higher concentrations than those used in clinical practice [11]. However, the clinical relevance of this inhibition is not established. Sevoflurane is an isopropylether volatile anesthetic which enhances the GABAergic transmission. Its effect on NMDA pathway has been demonstrated, with an inhibition of NMDA-gated currents and NMDA-induced mitochondrial membrane depolarization [12,13]. Sufentanil and atracurium fail to affect NMDA-R. Thus we hypothesize that anesthesia using both propofol and sevoflurane may have facilitated the inhibition of NMDA pathway, hence worsened the clinical presentation of anti-NMDA-R encephalitis. Although propofol was also used before surgery in our patients, its effect on NMDA-R might not have been sufficient in itself to worsen neurologic symptoms.

Several case series of anti-NMDA-R encephalitis advocate surgery to remove teratoma, but fail to provide detailed anesthetic procedure or to describe the postoperative course. Pascual-Ramirez et al. [2] reported two cases without any complication of anesthesia (propofol/sevoflurane/fentanyl and propofol/remifentanil, respectively). Several reasons could explain the difference of results between these two cases and ours. The dosages of anesthetic drugs used in the two cases are not reported and may have been lower than in our case. For the first patient propofol was not used after the procedure. For the second patient plasmapheresis was fulfilled before surgery and could have been more efficient than IVIg to rapidly decrease anti-NMDA-R titers in serum. Sevoflurane was also not used. Kawano et al. [9] reported another patient with a suspected limbic encephalitis who underwent a teratoma resection without complication (remifentanil, thiamylal and sevoflurane) but anti-NMDA-R level was not determined. Unlike for our patient propofol was not used. Splinter et al. [14] reported the occurrence of a rapid and profound hypotension with propofol used for a diagnostic lumbar puncture performed in a 14-year-old female at the time of diagnosis. Ten months later, another lumbar puncture was performed under propofol and sevoflurane without adverse reaction. Dalmau et al. [1] also reported a pronounced hypotension secondary to propofol injection at induction of anesthesia for a lumbar puncture. The same procedure was performed several months later with a reduced dose of propofol and no hypotension was noted that time.

Our observation highlights the specificities of anesthetic management in patients with anti-NMDA-R encephalitis. Well-known NMDA antagonists such as nitrous oxide, xenon, ketamine, tramadol and dextropropoxyphene should be avoided. Nevertheless, many other drugs that can inhibit NMDA-R, such as sevoflurane and propofol, could potentiate each other. Accordingly, the inappropriate use of certain anesthetic drugs must be suspected when the neurological status worsens after a surgical procedure in patients with anti-NMDA-R encephalitis. Anesthesia using benzodiazepines, opiates and curares, which fail to interfere with the NMDA pathway, should be preferred in these patients.

### Consent

Written informed consent was obtained from the patient for publication of this case report and of the videos associated. A copy of the written consent is available for review by the Editor of this journal.

## Abbreviations

NMDA-R: N-methyl-D-aspartate receptor; CSF: Cerebrospinal fluid; PCR: Polymerase chain reaction; HSV: Herpes simplex virus; VZV: Varicella Zoster virus; EEG: Electroencephalogram; MRI: Magnetic resonance imaging; ICU: Intensive care unit; IVIg: Intravenous immunoglobulins; AMPA-R: α-amino-5-methyl-3-hydroxy-4-isoxazole propionic acid receptor.

## Competing interests

The authors declare that they have no competing interests.

## Authors’ contribution

FXL, LM, BF 1) acquisition and interpretation of data 2) article drafting 3) approved version. CK, FP, JH, NP, PV 1) acquisition of data 2) article revision 3) approved version. All authors read and approved the final manuscript.

## Pre-publication history

The pre-publication history for this paper can be accessed here:

http://www.biomedcentral.com/1471-2253/14/5/prepub

## Supplementary Material

Additional file 1 It shows the patient with dyskinesia. Click here for file

Additional file 2 It shows the patient with worsened dyskinesia. Click here for file
